# Upper Esophageal Perforation with Cervico-Mediastinal Extension Successfully Treated with Endoluminal Vacuum Therapy: A Case Report Highlighting Inflammatory Marker Dynamics

**DOI:** 10.3390/life16040639

**Published:** 2026-04-10

**Authors:** Bogdan Mihnea Ciuntu, Edwina-Elena Viciriuc, Andreea Ludusanu, Adelina Tanevski, Mihaela Corlade-Andrei, Sorin Nicolae Peiu, Raluca Alina Dragomir, Dan Vintila, Cristinel Ionel Stan, Gheorghe Balan

**Affiliations:** 1 Department of General Surgery, Grigore T. Popa University of Medicine and Pharmacy Iasi, 700115 Iasi, Romania; bogdan-mihnea.ciuntu@umfiasi.ro (B.M.C.); papancea.adelina@umfiasi.ro (A.T.); dan.vintila@umfiasi.ro (D.V.); 2 Faculty of Medicine, Grigore T. Popa University of Medicine and Pharmacy Iasi, 700115 Iasi, Romania; raluca.dragomir@umfiasi.ro; 3 Department of Morpho-Functional Sciences I—Anatomy, Grigore T. Popa University of Medicine and Pharmacy Iasi, 700115 Iasi, Romania; cristinel.stan@umfiasi.ro; 4 Department of Emergency Medicine, Surgery II, Grigore T. Popa University of Medicine and Pharmacy Iasi, 700115 Iasi, Romania; mihaela.corlade2@umfiasi.ro; 5 Department of Vascular Surgery, Grigore T. Popa University of Medicine and Pharmacy Iasi, 700115 Iasi, Romania; sorin-nicolae.peiu@umfiasi.ro; 6 Department of Gastroenterology, Grigore T. Popa University of Medicine and Pharmacy Iasi, 700115 Iasi, Romania; gheorghe-g-balan@umfiasi.ro

**Keywords:** cervicomediastinitis, cervico-mediastinal abscess, cervical esophagus, cervical esophageal perforation, EVAC, deep neck infection, tracheostomy

## Abstract

**Background:** Upper esophageal perforations are life-threatening conditions associated with a high risk of mediastinitis, sepsis, and multiorgan failure. Standard management often requires extensive surgical intervention, which carries substantial morbidity. **Methods:** We report the case of a 56-year-old male with an iatrogenic cervical esophageal perforation complicated by cervicomediastinal abscess formation. Due to anatomical constraints preventing standard endoluminal approaches, a hybrid organ-preserving strategy was employed, consisting of surgical drainage combined with an externally adapted vacuum-assisted closure (VAC) system applied adjacent to the esophageal defect. **Results:** The patient demonstrated progressive clinical improvement without the need for esophageal diversion or major reconstructive surgery. Inflammatory markers were monitored serially and showed a downward trend, serving as adjunctive indicators of treatment response. The esophageal defect healed successfully, was confirmed radiologically, and no treatment-related complications were observed. **Conclusions:** This case suggests that externally adapted VAC therapy may represent a potential organ-preserving option in selected patients with complex cervical esophageal perforations when conventional techniques are not feasible. Further studies are required to validate this approach.

## 1. Introduction

Esophageal perforation is a rare but life-threatening condition, with reported mortality rates ranging from 20% to 40%, depending on the location of the injury, etiology, and timing of diagnosis and intervention [1]. Cervical and proximal esophageal perforations are less common than distal injuries but pose distinct clinical challenges due to their proximity to critical neurovascular structures and the high risk of rapid extension into the mediastinum [2].

Esophageal perforation represents a surgical emergency, particularly when diagnosis or treatment is delayed, significantly increasing the risk of mediastinitis, sepsis, and multiorgan failure [3,4]. Although thoracic perforations occur more frequently, cervical esophageal leaks are especially complex to manage because of anatomical constraints and the potential for cervicomediastinal spread of infection [5,6]. Traditionally, management has relied on surgical approaches ranging from primary repair to esophageal diversion or esophagectomy, all of which are associated with substantial morbidity [3,6].

In recent years, minimally invasive endoscopic techniques have emerged as viable alternatives in selected patients. Among these, endoluminal negative pressure therapy (EndoVAC) has gained increasing recognition for the management of gastrointestinal leaks and perforations, particularly in high-risk patients unsuitable for extensive surgery [4,7]. EndoVAC promotes healing by applying continuous negative pressure to the defect site, facilitating effective drainage, reducing local edema, and stimulating granulation tissue formation, ultimately leading to defect closure [7,8].

This article reports a clinical case of an upper esophageal perforation complicated by cervicomediastinitis, managed using a modified vacuum-assisted closure (VAC) system adapted for external application to the esophageal fistulous defect. In this case, traditional endoluminal approaches, including stenting and conventional EndoVAC placement, were not feasible. The externally adapted VAC system allowed for controlled deep cervical drainage, enhanced granulation, and supported progressive closure of the esophageal wall from the outside. Additionally, this report highlights the clinical utility of monitoring inflammatory marker trends to guide VAC system exchanges and assess treatment response.

## 2. Case Presentation

A 56-year-old male presented on day 0 (admission) with dysphagia, inspiratory dyspnea, cervical swelling, subcutaneous emphysema, and fever. His medical history included a septoplasty performed 4 days prior, with a high likelihood of iatrogenic esophageal injury during endotracheal intubation. Laboratory investigations on admission revealed leukocytosis with neutrophilia and markedly elevated inflammatory markers, including CRP, procalcitonin, and presepsin, consistent with a systemic inflammatory response and a high risk of severe infection or sepsis (Table 1).

**Figure 1:**
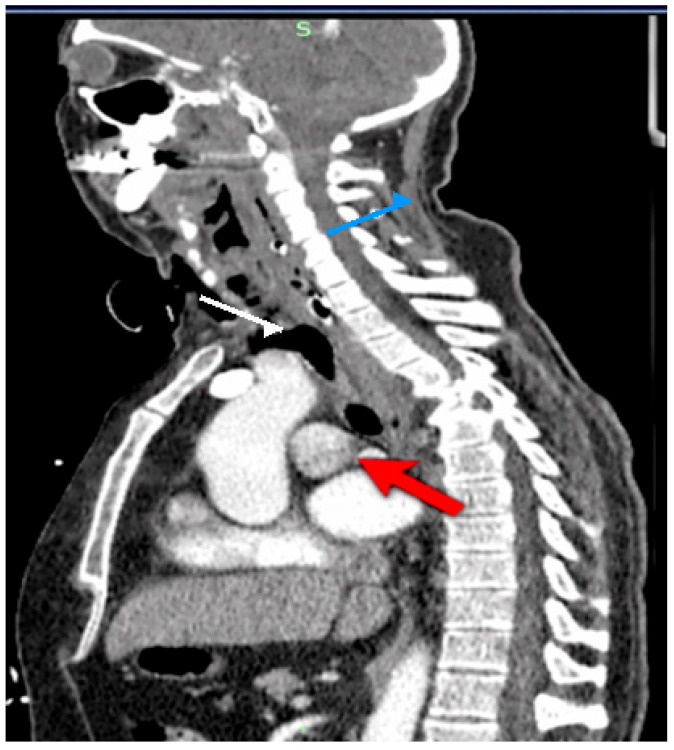
Contrast-enhanced CT scan (sagittal view) demonstrating a right paraesophageal fluid collection with associated gas bubbles and mediastinal extension. The **red arrow** indicates the paraesophageal fluid collection, the **white arrow** indicates intralesional gas bubbles, and the **blue arrow** indicates extension into the mediastinum, consistent with cervicomediastinal abscess formation secondary to esophageal perforation.

CT imaging showed extensive cervical emphysema with fascial spread, associated retropharyngeal and paraesophageal inflammation, and a right paraesophageal mediastinal collection (~40 mm) extending cranially as a large retropharyngeal air–fluid collection. Pneumomediastinum was also present (Figure 1).

Flexible esophagoscopy identified a fistula approximately 2 cm below the upper esophageal sphincter (UES), measuring 12 × 4 mm.

In this patient, the fistula was located very close to the upper esophageal sphincter, at a level where standard endoluminal sponge positioning would have been technically unstable and poorly tolerated. The proximity to the pharyngoesophageal junction increased the risk of device displacement, ineffective sealing, and interference with the already compromised upper airway. In this context, an externally adapted approach offered a more controllable means of cavity drainage and local defect management.

**Figure 2:**
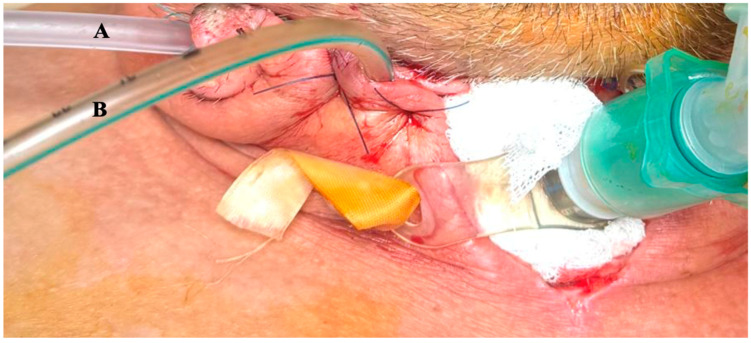
(**A**). Drainage tube. (**B**) Double lumen VAC adapted externally and placed close to the esophageal defect.

**Figure 3:**
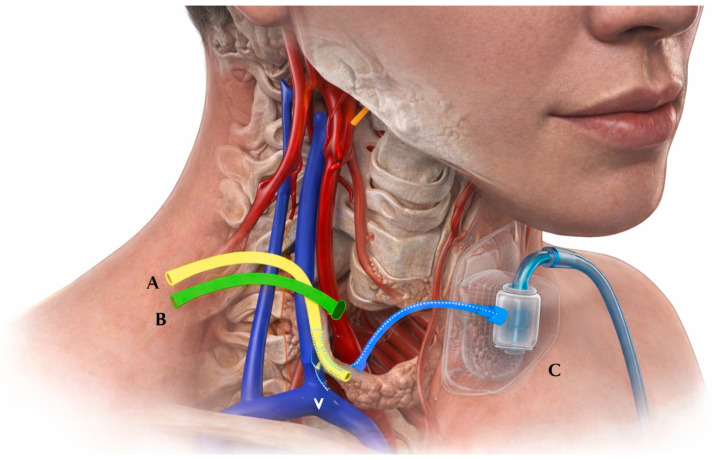
Patient-specific schematic based on CT imaging. (**A**) Fistula location; (**B**) drainage trajectory toward the internal jugular vein; (**C**) externally adapted VAC system positioned over the fistula, adapted from a reference image in Complete Anatomy (3D4Medical from Elsevier).

A hybrid approach was adopted: tracheostomy and airway stabilization–Cervicotomy with drainage–Thoracotomy with mediastinal lavage–Placement of external VAC system. The externally adapted VAC system consisted of a double-lumen drainage tube (medical-grade silicone, approximately 14–16 Fr)–Polyurethane sponge tailored to cavity size–Continuous negative-pressure suction (−150 to −170 mmHg) (Table 2). The sponge was positioned adjacent to the esophageal defect via the cervical incision, ensuring direct contact with the infected cavity. A semi-occlusive dressing system was used to maintain an airtight seal. The system was secured using sutures and adhesive dressings (Figure 2 and Figure 3).

Local irrigation was performed using sterile saline solution (20–50 mL per session), once daily, through the secondary lumen to assist with debris clearance and infection control. Inflammatory markers (CRP in mg/L, WBC ×10^3^/μL, procalcitonin ng/mL) were monitored daily.

Postoperatively, the patient was managed in the intensive care unit with mechanical ventilation, targeted antimicrobial therapy, enteral nutrition via a nasogastric tube, and serial radiological monitoring. Cervical wound care and drainage output were monitored daily, and the tracheostomy cannula was maintained with regular suction and periodic replacement.

Microbiological cultures identified *Enterobacter cloacae* and *Lactobacillus rhamnosus*, with antibiotic therapy adjusted accordingly. A subsequent multidrug-resistant *Acinetobacter baumannii* infection required treatment with colistin, resulting in successful infection control.

Inflammatory markers were monitored serially as adjunctive indicators of treatment response. A pattern of gradual decline followed by transient increases was observed, supporting—but not solely determining—the timing of VAC system exchanges until complete defect closure.

**Figure 4:**
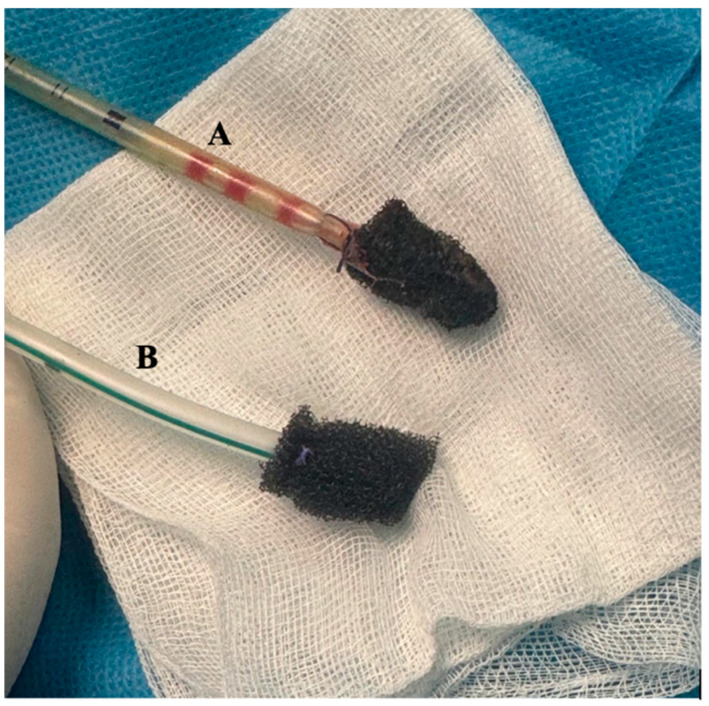
(**A**) removed VAC (after 6 days of use); (**B**) new VAC (to be placed).

**Figure 5:**
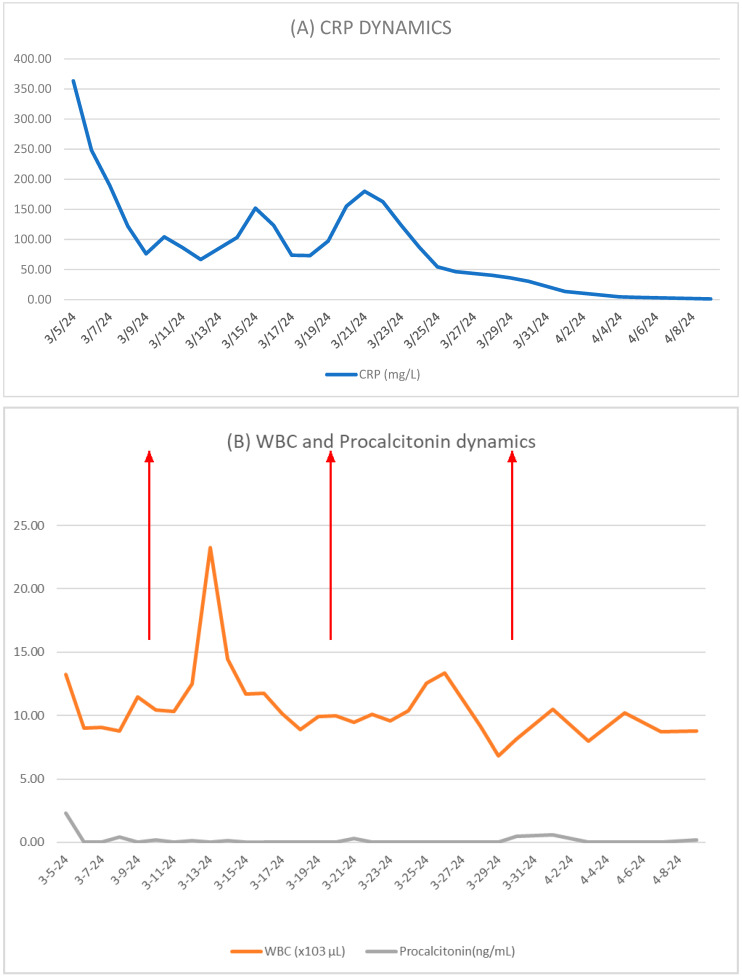
Temporal evolution of inflammatory markers during treatment. (**A**) C-reactive protein (CRP, mg/L) levels. (**B**) White blood cell count (WBC, ×10^3^/μL) and procalcitonin (ng/mL). A general downward trend is observed, with transient fluctuations during therapy. Red arrows indicate VAC system exchange time points.

VAC system exchanges were generally performed at intervals of approximately 6–7 days (Figure 4). However, the exact timing was not based on a predefined biomarker threshold, but rather on combined assessment of local findings, imaging, overall clinical status, and inflammatory marker trends. In this context, the observed pattern of progressive decline followed by transient re-elevation in inflammatory parameters was considered a useful adjunctive indicator supporting the timing of reassessment and replacement (Figure 5).

**Figure 6:**
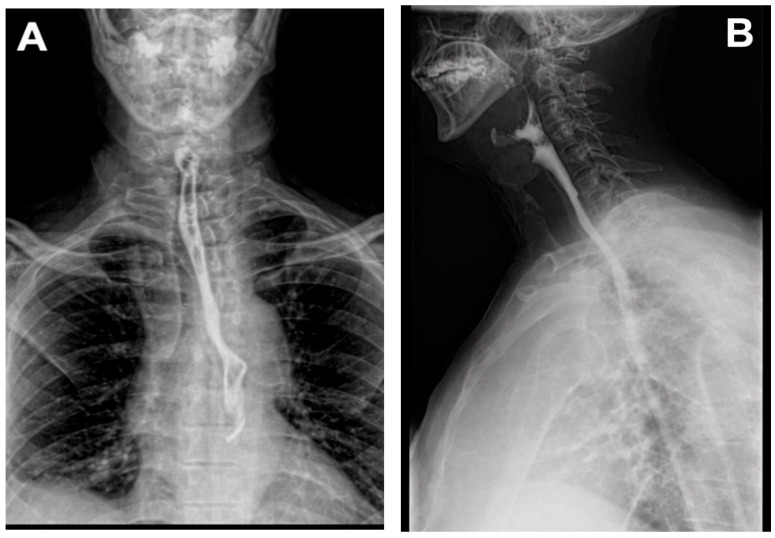
(**A**,**B**) Barium swallow test radiography showing continuous flow through the esophagus.

After more than four weeks of treatment using the system, contrast-enhanced radiologic imaging revealed a normal esophageal appearance with no evidence of leakage, confirming successful closure of the defect (Figure 6). The patient was discharged following an extended hospitalization, in improved general condition, with recommendations for routine follow-ups. The timeline of clinical events, including surgical management and sequential VAC system exchanges, is summarized in Table 3, illustrating the progressive clinical improvement and eventual defect closure.

## 3. Discussion

The patient presented with a high cervical esophageal fistula located approximately 2 cm below the upper esophageal sphincter, raising suspicion of prior iatrogenic injury, possibly related to orotracheal intubation. Although rare, esophageal perforation secondary to endotracheal intubation has been documented in the literature [9,10], the contemporary literature continues to identify iatrogenic injury as the most frequent cause of esophageal perforation, with delayed or subtle clinical presentation being common, especially in cervical lesions [11]. Recent analyses highlight that a significant proportion of patients present beyond 24 h from injury, underscoring the importance of maintaining a high index of suspicion when evaluating proximal esophageal defects in the context of recent airway or upper gastrointestinal interventions [12].

This case highlights the effectiveness of vacuum-assisted closure (VAC) therapy in managing upper esophageal perforation with cervicomediastinal involvement, conditions that traditionally require aggressive surgical intervention [13]. VAC therapy offers several advantages: local control of infection and leak without esophageal diversion, promotion of rapid granulation and tissue repair, and preservation of the native esophagus, thereby avoiding the morbidity associated with cervical esophagostomy or esophagectomy [14]. A recent 2024 systematic review and meta-analysis reported a pooled clinical success rate of approximately 88% for EVAC in esophageal defects, including transmural perforations, with relatively low associated mortality [15]. These findings reinforce the role of vacuum-assisted therapy as a viable alternative to more invasive surgical strategies, particularly in selected patients.

Emerging data from 2025 further support this paradigm shift. Comparative analyses between EVT and esophageal stenting demonstrate that both modalities achieve high rates of defect closure; however, EVT appears to be associated with lower complication rates and shorter treatment durations in several studies [16]. Endoluminal negative-pressure aspiration has shown promise for treating fistulas in the upper gastrointestinal tract [16,17]. Modifying the system to include a double-lumen design allows for simultaneous lavage and improved sealing of the defect. Advantages include the potential for endoscopic placement under moderate sedation, reduced requirement for general anesthesia and intubation, decreased device manipulation, and improved patient tolerance. Esophageal stenting remains a non-surgical alternative; however, stent migration, persistent leakage, and risk of esophagotracheal fistula formation have been reported [17]. Endoscopic vacuum therapy (EVAC) may represent a potential organ-preserving therapeutic option in selected cases, with strategic sponge and drain placement supporting continuous drainage (typically 125 mmHg, not exceeding 175 mmHg), promoting progressive closure of the defect [18,19].

In this patient, the fistula was located very close to the upper esophageal sphincter, at a level where standard endoluminal sponge positioning would have been technically unstable and poorly tolerated. The proximity to the pharyngoesophageal junction increased the risk of device displacement, ineffective sealing, and interference with the already compromised upper airway [20,21]. The externally adapted VAC system retained the principles of negative-pressure therapy while positioning the sponge outside the esophageal wall via a lateral cervical incision. The sponge was custom-sized to fit the lesion and initially placed in direct contact with the defect, then gradually retracted toward the periphery. This approach ensured thorough suction of the cavity, enhanced local microvascular perfusion, and facilitated gradual closure along the distal tip during subsequent repositioning. The tissue repair process mirrored that of endoluminally treated lesions [22]. Additionally, two-lumen lavage systems can remain in situ for up to seven days, minimizing the need for repeated anesthesia.

Monitoring inflammatory markers provided valuable insight into infection control and therapeutic response. Serial measurements of CRP, WBC, and procalcitonin allowed for the non-invasive assessment of clinical progress and guided VAC exchanges. In this patient, inflammatory markers gradually decreased over 4–5 days, followed by a transient rise over 1–2 days, indicating bacterial accumulation in the sponge and prompting device exchange. This recurring pattern persisted until complete closure of the esophageal defect, coinciding with normalization of inflammatory parameters.

VAC techniques have proven effective for enteral fistulas by promoting healing and managing local drainage. However, their high cost, care requirements, and need for a multidisciplinary team must be considered. In this case, the externally adapted VAC system provided deep cervical drainage, enhanced infection control, facilitated secretion clearance, and accelerated tissue repair [23]. Despite these benefits, EndoVAC therapy requires specialized expertise, endoscopic access, and careful monitoring. Further studies are needed to establish standardized protocols and compare long-term outcomes with traditional surgical approaches.

This report has several limitations. First, it describes a single clinical case, which limits the generalizability of the findings. Second, the externally adapted VAC technique was applied in a highly specific anatomical and clinical context and may not be reproducible or appropriate in all esophageal perforations. Third, although inflammatory marker dynamics appeared clinically useful in this case, their role in guiding VAC exchanges remains observational and should not be interpreted as validated evidence. Further experience in larger case series is required to better define the indications, technical reproducibility, and outcomes of this approach.

## 4. Conclusions

Effective management of surgical complications requires not only targeted intervention but also comprehensive assessment of the patient’s overall physiological status. Optimal outcomes are achieved through a multidisciplinary approach involving surgeons, anesthesiologists, gastroenterologists, and radiologists. Endoluminal vacuum-assisted closure (EVAC) has increasingly been recognized as an adjunctive technique for the treatment of anastomotic leaks and gastrointestinal fistulas. Continuous clinical monitoring and structured follow-up protocols are essential for early detection of therapeutic failure and timely intervention.

This case demonstrates that complex cervical esophageal fistulas, previously considered inoperable or anatomically inaccessible, can be successfully managed using externally applied VAC therapy tailored to the lesion’s specific anatomy. This approach provides a minimally invasive, organ-preserving, and may represent a promising therapeutic option in selected cases, alternative to conventional surgical strategies. Furthermore, serial monitoring of inflammatory biomarkers proved valuable in guiding therapy and assessing clinical response throughout the course of treatment. However, conclusions are limited by the single-case nature of this report, and further studies are needed to establish its safety, efficacy, and reproducibility.

## Figures and Tables

**Table 1:** Laboratory parameters on admission.

*Laboratory Investigations*	*Values*	*Normal Values/Interpretation*
*Leucocytes*	*13.25 × 10*^3^/μL	*4.0–10.0 × 10*^3^ μL
*C-reactive protein*	*3625* mg/L	*0–5 *mg/L**
*Procalcitonin*	*2.30 *ng/mL**	*2–10 *ng/mL* indicated high risk of severe infection*
*Presepsin*	*1069* pg/mL	*>1000 *pg/mL* indicates high risk of sepsis*
*Neutrophils*	*11.85 × 10*^3^/μL	*2.0–8.0 × 10*^3^/μL
*Lymphocytes*	*0.75 × 10*^3^/μL	*1.0–4.0 × 10*^3^/μL
*Hemoglobin*	*11.4* g/dL	*13.0–17.3* g/dL
*Hematocrit*	*33*%	*39–51*%
*Urea*	*68* mg/dL	*13–43* mg/dL
*Potassium*	*3.0* mmol/L	*3.5–5.1* mmol/L
*Alkaline reserve*	*26.9* mmol/L	*22–29* mmol/L

**Table 2:** Key settings and adjunctive care in Vacuum Therapy.

*Parameter*	*Value/Range*
*Vacuum pressure applied*	*−150 *mmHg*–170* mmHg
*Sponge replacement interval*	*Every 6 or 7* days
*Endoscopic confirmation of placement*	*Required at every replacement*
*Nutritional support*	*Enteral (Via nasogastric tube)*
*Additional therapy*	*Broad-spectrum antibiotics*

**Table 3:** Timeline of clinical events.

Day	Clinical Event
Day 0	Admission, CT scan, diagnosis
Day 1	Cervicotomy, thoracotomy, drainage, VAC placement
Day 5–7	First VAC exchange
Day 10–14	Second VAC exchange
Day 20+	Third VAC exchange Progressive clinical improvement
~Day 30	Closure confirmed (contrast study)
Discharge	Patient discharged

## Data Availability

All original contributions from this study are presented in the article. Please contact the corresponding author for any further inquiries.
